# Developing a clinical decision tool based on electroretinogram to monitor the risk of severe mental illness

**DOI:** 10.1186/s12888-022-04375-3

**Published:** 2022-11-18

**Authors:** Rossana Peredo, Marc Hébert, Chantal Mérette

**Affiliations:** 1 Department of Social and Preventive Medicine, Faculty of Medicine, Laval University, Quebec, QC Canada; 2 CERVO Brain Research Centre, Quebec, QC Canada; 3 Department of Ophthalmology and otorhinolaryngology, Faculty of Medicine, Laval University, Quebec, QC Canada; 4 Department of Psychiatry and Neuroscience, Faculty of Medicine, Laval University, Quebec, QC Canada

**Keywords:** Biomarker, Bipolar disorders, Schizophrenia, Early detection, Electroretinography

## Abstract

**Background:**

We have shown that electroretinograms can discriminate between patients with severe mental illness (SMI) and healthy controls in previous studies. We now intend to enhance the development and clinical utility of ERG as a biological tool to monitor the risk of SMI.

**Methodology:**

A sample of 301 SMI patients (bipolar disorder or schizophrenia) and 200 controls was first split into a training (*N* = 401) and testing dataset (*N* = 100). A logistic regression using ERG was modeled in the training data, while external validation and discriminative ability were assessed in the testing data. A decision curve analysis was used to test clinical usefulness. Moreover, the identification of thresholds of uncertainty based on the two-graph ROC and the interval of uncertainty was used to enhance prediction.

**Results:**

The discriminative assessment of the ERG showed very high sensitivity (91%) and specificity (89%) after considering uncertainty levels. Furthermore, for prediction probabilities ranging from 0.14 to 0.95 in the testing data, the net benefit of using our ERG model to decide whether to intervene or not exceeded that of never or always intervening.

**Conclusion:**

The ERG predicted SMI risk with a high level of accuracy when uncertainty was accounted for. This study further supports the potential of ERG to become a useful clinical decision tool to decide the course of action for subjects at risk of SMI. However, further investigation is still needed in longitudinal studies to assess the external validity of the instrument.

**Supplementary Information:**

The online version contains supplementary material available at 10.1186/s12888-022-04375-3.

## Introduction

The importance of early detection of a severe mental illness (SMI), such as schizophrenia or bipolar disorder, is widely recognized. Substantial literature suggests that prompt interventions improve the clinical outcome of individuals with psychotic symptoms and may even prevent or at least delay the appearance of psychosis [1–3]. However, there are currently no “gold standard” instruments to identify the appearance of SMI. Two clinical instruments have been widely recognized for early detection: the Comprehensive Assessment of at-Risk Mental States (CAARMS) [4] and the Structured Interview for Prodromal Syndromes (SIPS) [5]. It has been reported that the transition rate to psychosis among individuals identified as high-risk according to these instruments is approximately 36% after 3 years of follow-up [6]. Although these clinical syndromes are clearly sensitive in detecting susceptibility to developing SMI, the instruments lack specificity, and a large percentage of high-risk individuals will not transit to a full psychotic episode or will possibly rather present poor functional outcomes or other comorbid mental disorders [7]. Moreover, those labeled as high-risk who do not transit will bear the burden of psychiatric stigma and/or may receive inappropriate care. Therefore, further work should be done to establish a detection system of SMI that increases the accuracy of disease prediction so that it minimizes the risk of unnecessary stigmatization and enables clinicians to offer appropriate management according to the needs of each individual.

Another challenge is that diagnostic instruments for SMI are based on symptomatic criteria depending mainly on patients’ reports. Unlike other diseases such as cancer, where diagnosis and prognosis assessments rely on specific biomarkers, there is a lack of biological tests approved for clinical use [8] in psychiatry. This hampers efforts to define reliable clinical phases of psychotic illnesses [9], thereby complicating the implementation of appropriate screening and monitoring approaches. Hence, an increasing number of researchers are turning their interest to developing accurate biomarkers for mental health diseases such as SMI.

Among potential biomarkers, the retina has gained particular interest in recent years, due to its common embryonic origin with the brain suggesting that structural and functional changes in this organ may be aligned with some retinal changes [10, 11]. The electroretinogram (ERG), a very well-known instrument commonly used to assess the functional electrical response of retinal photoreceptors (i.e., rods for scotopic vision and cones for photopic vision [12]), has been shown to be a promising tool to identify SMI given that ERG anomalies in patients with psychotic disorders were found in several studies [13–15]. Moreover, our research team has already reported very high accuracies when distinguishing patients from healthy control subjects (91% for schizophrenia and 89% for bipolar disorder) using ERG measurements [16]. Our previous studies also reported ERG anomalies even in young offspring at genetic risk of SMI [17], and we observed an association between ERG anomalies and cognitive impairment in offspring at an early age before the appearance of symptoms [18].

The present study aimed to enhance the development of the ERG as a biological tool to monitor susceptibility to SMI. For this purpose, a predictive regression model was developed using ERG measures. Only photopic (cone) responses were included in the model given that in addition to the effectiveness of the ERG as a biological tool, this time we are interested in the efficiency of its clinical use (the photopic ERG necessitates only 10 minutes of light adaptation in contrast to the scotopic ERG which necessitates 20–30 min of dark adaptation). Because our focus was to predict a vulnerability of SMI before the occurrence of first symptoms, whether it is for schizophrenia or bipolar disorder, both diseases were grouped together and considered as SMI. Also, given the recognized genetic overlap between schizophrenia and bipolar disorder they may share susceptibility [19, 20]. Since special attention should be given to the harms of false-positive individuals—who may be unnecessarily targeted—and false-negative individuals—who might miss further monitoring, the clinical utility of the ERG regression model was assessed using a technique that proposes a “net benefit” value that gives a different ponderation to true and false-positives [21]. Additionally, three levels of certainty (i.e., probable SMI, uncertain, and no disease) instead of two were established so that the third category of uncertainty would identify individuals for whom the ERG may be inconclusive. Hence, the uncertain intermediate level will not immediately receive a psychiatric label but will still benefit from further monitoring.

## Methods

### Data source and study population

This is a cross-sectional study approved by the Neuroscience and Mental Health Research Ethics Committee of our institution (CIUSSS Capitale-Nationale). The database was previously analyzed to show the high accuracy of ERG prediction [16]. However, because the present objective is to enhance the development of a preliminary screening instrument to detect susceptibility to SMI, whether it is schizophrenia or bipolar disorder, subjects with the two diagnoses were combined, obtaining a total sample of *N* = 301 SMIs who were unrelated and stabilized outpatients*.* Participants were referred by their treating psychiatrists from a university hospital or the regional psychiatric department from Quebec City and Beauce region of the Province of Quebec. Inclusion criteria were having a diagnosis of schizophrenia or bipolar disorder according to the DSM-IV criteria, being between 21 and 55 years old, and having normal vision with no known ocular pathology. Exclusion criteria were having brain and metabolic disorders, being pregnant, having used drugs including cannabis in the past 24 hours, having traveled two time zones within 1 month before the experiment, and working on night shift (which could disrupt the retinal internal clock) [17, 22].

As detailed in our previous work [16] healthy control subjects were recruited through advertisements from the same population of Quebec. Exclusion criteria were the same as for patients, with the addition of having any Axis I DSM-IV diagnosis and having a positive family history of schizophrenia, bipolar disorder, or major depressive spectrum disorders. Signed consent was obtained for all participants.

### ERG measurements included as predictors in the regression model

ERG recordings were performed in nondilated eyes as per Gagne et al., 2010 [23] using Espion (E2, E3) Systems and color dome ganzfeld (Diagnosys LLC, Lowell, MA) with a background set at 80 cd/m2 and recording from both eyes was achieved with a DTL electrode placed into the conjunctival sac. The reliability and reproducibility of the ERG protocol and acquisition techniques used in this study have been extensively demonstrated [23–25]. Further details about the protocol can be found in an earlier publication [16]. Briefly, two components of a typical ERG waveform were measured: the a- and b-waves. For each component, two parameters were registered: the amplitude (amp) and the latency (lat). Each of these parameters was measured at two steps: at a fixed luminance of 7.5 cd•s/m2 and at V_max_ (defined as an average of ERG responses obtained at luminances of 13.3, 23.7, and 50.0 cd•s/m2 as per Hébert et al. 2017) [26]. ERG technicians were blinded to the participants’ diagnosis. In addition, the acceptability to participate in the study and to be assessed with ERG was very high (95%) among affected participants.

To enhance the clinical usefulness of ERG, which has already been shown to be a potential biomarker [16], special attention was given to the practicality and ease of use of the instrument. Because the cone ERG assessments requires a light adaptation of only 10 minutes, as opposed to the rods assessment which requires 20 to 30 minutes of dark adaptation, two logistic regression models were developed a priori (using the backward and forward stepwise method). One model included both cone + rod ERG parameters measurements and the other included only cone ERG parameters measurements. Both models yielded almost identical performance accuracies (see Fig. S1 in the supplements); thus, only the cone ERG parameters were used in this study as an attempt to minimize the discomfort of participants.

### Statistical analysis

All statistical analyses were conducted in R version 4.0.3. The first portion of the analysis was the development of the regression model (using the glm [27] and the stepAIC functions [28]). For this, the total sample was randomly split using an 80:20 ratio into a training dataset (241 SMI and 160 healthy controls) and a testing dataset (60 SMI and 40 healthy controls). A logistic regression model was then developed in the training dataset to predict the clinical status (SMI vs. control), using cone ERG measurements as predictive variables. The covariates pupil size, age, and sex were selected according to our previous publications [22, 26, 29, 30]. The backward and forward stepwise method was applied for variable selection. All regression model assumptions were adequately met such as independence, normality and no multicollinearity or extreme outliers.

In the second portion, the quality of the predictive ERG model was evaluated. For this, model stability and possible overfitting were assessed using leave-one-out cross-validation with the caret package [31]. Additionally, internal validation was evaluated by estimating the apparent performance (in the training dataset) using two indicators: Nagelkerke’s R2 and the Brier score. Calibration was assessed visually and with the Hosmer–Lemeshow test as an indicator. Then, external validation and the discriminative ability (represented with the area under the ROC curve {AUC-ROC}) were evaluated using the test dataset. The following study follows the TRIPOD statement criteria for reporting a prediction model [32, 33].

The third portion of the analysis assessed the clinical utility of the regression model using the decision curve analysis technique [34, 35]. Under this technique, a “net benefit” value is calculated using Formula 1, where pt. represents a threshold probability of developing SMI and *n* is the total sample.

1 $$\textrm{Net}\kern0.5em \textrm{Benefit}\kern0.5em =\kern0.5em \frac{\textrm{True-Positive}\kern0.5em \textrm{Count}}{\textrm{n}}\hbox{-} \frac{\textrm{False-Positive}\kern0.5em \textrm{Count}}{\textrm{n}}\left(\frac{\textrm{pt}}{1\hbox{-pt}}\right)$$

By providing weight to the false-positives based on pt., it is possible to represent a theoretical relationship between pt. of the predicted disease and the relative value of false-positive and false-negative results. Then, to interpret the potential clinical value of the regression model, two other extreme net benefit values are calculated for two hypothetical clinical situations [36]: 1. All participants are positive (hence, 0 false-positives), so they all receive further intervention, and 2. All are negative (hence, 0 true and false-positives), so no intervention is offered. The optimal strategy will be the one with the highest net benefit value. This technique assumes that pt. represents the threshold at which a practitioner or a patient would decide to pursue a future intervention (e.g., early treatment or monitoring changing symptoms). Thus, a “reasonable range of risk threshold” will be defined; this “reasonable” range means that no one would reasonably use a pt. outside that range to decide upon treatment [36].

Finally, the final ERG regression model was applied to the testing dataset, and three levels of predictive certainty were established: 1. Most likely, SMI, 2. Uncertain, and 3. Most likely, no disease. Using these results, the following predictive accuracy measures were calculated: sensitivity, specificity, and accuracy. The cutoff values to define the three levels were obtained by comparing two trichotomization methods according to their accuracy measures. The first method is a modified ROC analysis called two graph ROC (TG-ROC) [37]. This method selects the most certain ranges of model scores that are the best for use when deciding for or against a diagnosis. Therefore, two thresholds with a preselected sensitivity and specificity of 90% were established. As a result, an intermediate or borderline range between the two thresholds was identified, and only the results outside the intermediate range were considered as certain. The second method is called the interval of uncertainty [38]. It defines an interval around the intersection where “health” and “disease” distributions are equal. To do so, an R function [38] counts the true negatives and false negatives for all possible decision thresholds that are lower than the intersection and counts the true positives and false positives for all the decision thresholds above the intersection. Then, it searches all possible lower and upper combinations and chooses uncertain intervals with specificities and sensitivities below a given value of 0.55; thereby, it defines the model scores that are better not to use for a diagnosis (i.e., the uncertain level).

## Results

Table 1 summarizes the clinical and demographic characteristics of the subjects and shows that no significant differences were found between the 301 patients with SMI and the 200 healthy controls. Fifty percent of the SMI subjects were diagnosed with bipolar disorder, while the other 50% had schizophrenia. Prescribed medications are also described in Table 1. There were no missing data for the ERG measures, and as expected, the unadjusted associations with the outcome (SMI) showed statistically significant relationships for most of the ERG parameters (i.e., a-wave amplitude, b-wave amplitude, and b-wave latency); further details are presented in Table S1 in the supplemental section.

**Table 1:** Clinical and demographic characteristics and comparison between SMI and control subjects

	SMI *n* = 301	Control *n* = 200	*p* value*
Age years mean (SD)	40.1 (9.9)	39.35 (10.3)	0.43
Male n (%)	182 (60.5)	124 (62.0)	0.73
Pupil size, mm mean (SD)	3.79 (1.14)	3.80 (1.01)	0.90
Diagnosis, n (%)			
Schizophrenia	150 (49.8)	–	–
Bipolar disorder	151 (50.2)	–	–
Duration of illness, years mean (SD)	26.9 (8.0)	–	–
Medication, n (%)			
Olanzapine	45 (14.9)	–	–
Quetiapine	77 (25.6)	–	–
Clozapine	49 (16.3)	–	–
Risperidone	55 (18.3)	–	–
Lithium	73 (24.3)	–	–
Aripiprazole	35 (11.6)	–	–
Synthroid	27 (8.9)	–	–

- The *p* value comparing the severe mental illness (SMI) and control groups is based on the t-test for continuous variables and chi-square for categorical variables

*The significance level was set to *α* =0.05

### Quality assessment of the predictive model

**Fig. 1:**
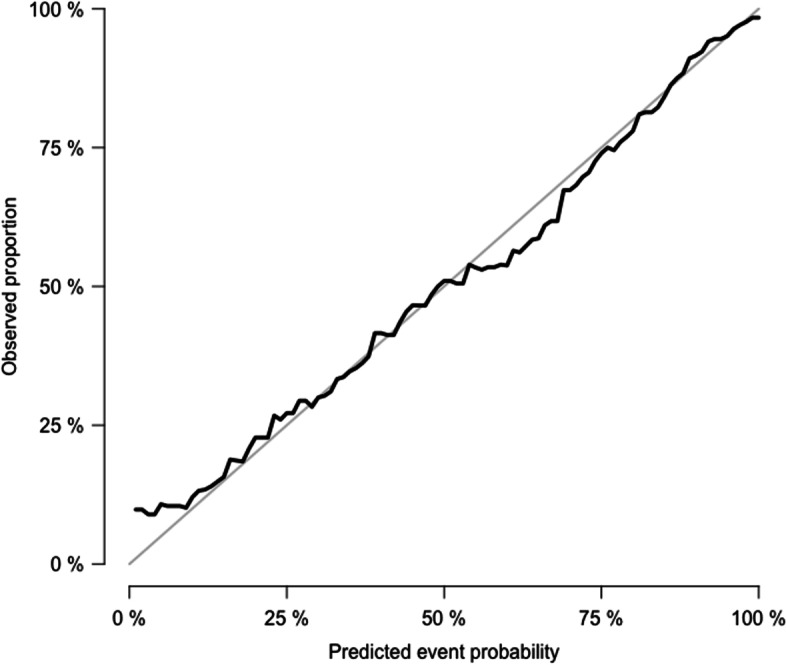
Calibration plot for the Best model, on the training data (*n* = 401)

The final best model yielded by the stepwise method included the following variables: a-wave amp fixed, b-wave lat fixed, b-wave lat Vmax and a-wave lat Vmax, age, and sex. The full model that includes all the variables is presented in Table S2 in the Supplements. Table 2 displays the internal validation of the best model showing that the apparent performance was good with a Brier score of 0.16 (1.0 would be the worst score) and a PseudoR2 Nagelkerkes score of 0.45 (the higher, the better). Visually, the calibration was good (see Fig. 1), and the Hosmer–Lemeshow test showed a *p* value of 0.74, which also indicates a good regression fit. The best model presented a high AUC-ROC of 0.85 and an accuracy of 0.77. All the parameters remained stable after leave-one-out cross validation with a Pseudo R2 of 0.41, a Brier score of 0.16, and a Hosmer–Lemeshow p value of 0.66.

**Table 2:** Model performance, discriminative ability, and internal and external validation

	Internal validation in the training data (*n* = 401)			External validation in the testing data (*n* = 100)
	Full model^a^ with all variables	Best model^b^	LOOCV	Best model^b^
Goodness of the fit				
Pseudo-R2 (Nagelkerkes) score	0.46	0.45	0.41	0.48
Brier Score	0.15	0.16	0.16	0.15
Hosmer–Lemeshow *p* value*	0.67	0.74	0.66	0.72
AIC score	399.03	392.51	–	–
Discriminative ability				
Accuracy	0.77	0.77	0.76	0.81
AUC [CI]	0.85 [0.81–0.89]	0.85 [0.81–0.88]	0.83 [0.79–0.87]	0.87 [0.80–0.94]

Note. – Model performance, discriminative ability, and internal validation including the full model and best model using leave-one-out cross validation (LOOCV) on the training dataset (*n*=401) and external validation including the best model using the testing dataset (*n*=100)

^a^: Full model: a-wave amp fixed + a-wave lat fixed + b-wave amp fixed + b-wave lat fixed + b-wave lat Vmax + a-wave amp Vmax + a-wave lat Vmax + b-wave amp Vmax + age at ERG + sex +pupil size

^b^: Best model: predictor variables were selected using the backward and forward stepwise method. The model selected: a-wave amp fixed + b-wave lat fixed + b-wave lat Vmax + a-wave lat Vmax + age at ERG + sex

*Significance levels set at 0. 05, *p* value >0.05 indicates no evidence of poor fit

*LOOCV* Leave-one-out cross validation with the variables of the best model. *AIC* Akaike information criterion, *AUC* Area under the ROC curve, *CI* Confidence Interval, *amp* amplitude, *lat* latency

External validation using the testing dataset is also presented in Table 2; interestingly, with this model, better performance measurements were found (Pseudo R2 squared of 0.48, Brier score of 0.15, and good calibration visually and statistically: *p* value = 0.72). The discriminative ability remained high, with an accuracy of 0.81 and AUC-ROC of 0.87 (CI: 0.80–0.94). Since medication may represent a confounder to consider in our results, a sensitivity analysis was performed, including medication in the final best model (see Table S3 in the supplements); the results remained robust and thus are not presented in this study. In addition, our previous publications showed no important impact of this variable in the regression analysis [16].

### Clinical utility of the regression model

**Fig. 2:**
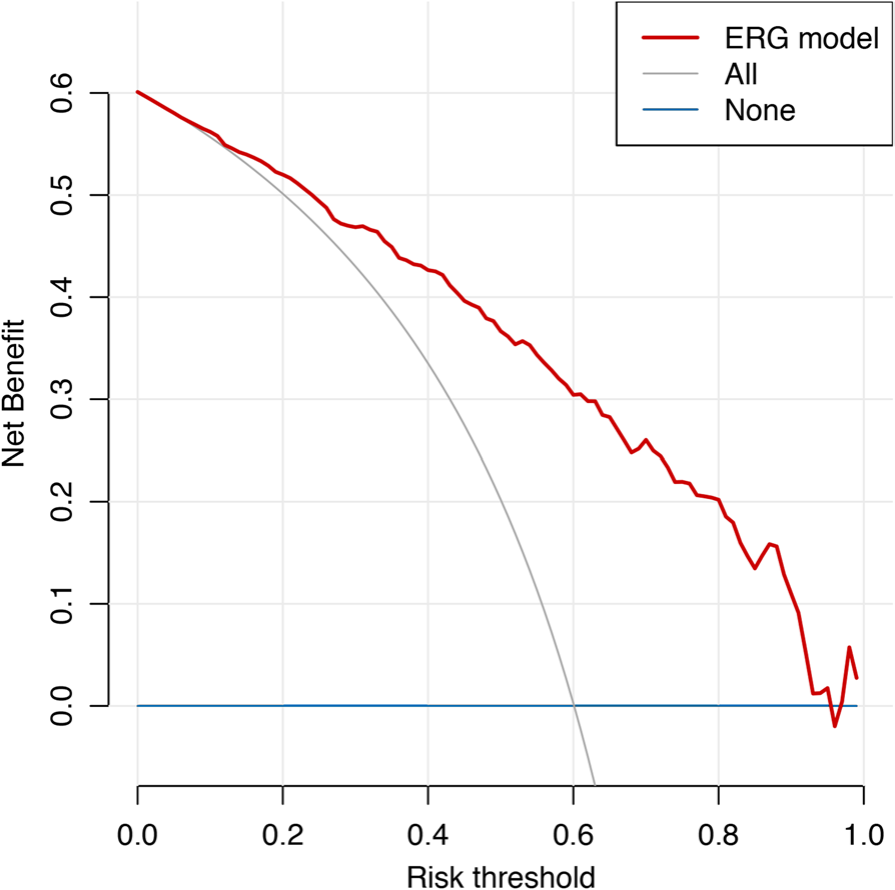
Clinical usefulness of the ERG regression model for SMI prediction: Decision curve. Note. Training data was used (*n* = 401). Clinical utility of the ERG regression model in terms of net benefit compared to provide intervention to all participants and none receives intervention

Figure 2 displays the decision curve analysis assessed on the testing dataset. This illustrates that the net benefit of using the ERG predictive model to make a clinical decision exceeded that of the hypothetical situation of intervening with all participants and exceeded the net benefit of no one receiving any intervention. The clinical utility of the ERG remained superior for predictions ranging from 0.14 to 0.95, which can be assumed to comprise the “reasonable range of risk threshold” [39] for most clinical practitioners.

The three levels of predictive certainty are presented in Table 3. The two trichotomization methods applied to the model using the testing dataset yielded very similar cutoff points with a very high predictive performance of > 0.89. The TG-ROC method performed slightly better (accuracy = 0.90, sensitivity = 0.91, and specificity = 0.89) than the uncertainty interval method (accuracy = 0.89, sensitivity = 0.89, and specificity = 0.88).

**Table 3:** Accuracy of the regression model according to the TG-ROC and uncertainty interval methods

Two-Graph ROC					Uncertainty Interval				
	Cutoff points 0.36 & 0.76	Observed				Cutoff points 0.41 & 0.73	Observed		
		SMI	Control	*Total*			SMI	Control	*Total*
Predicted	Most likely, SMI > 0.76	31_(a)_	2_(b)_	33	Predicted	Most likely, SMI >0.73	33_(a)_	3_(b)_	36
	Uncertain [0.36–0.76]	26	21	47		Uncertain [0.41–0.73]	23	15	38
	Most likely, not SMI < 0.36	3_(c)_	17_(d)_	20		Most likely, not SMI < 0.41	4_(c)_	22_(d)_	26
	*Total*	60	40	100		*Total*	60	40	100
	Sensitivity = 0.91	Specificity = 0.89				Sensitivity = 0.89	Specificity = 0.88		
	Accuracy = 0.90					Accuracy = 0.89			

Note. Accuracy of the regression model predicting severe mental illness (SMI) with ERG measures, allowing for an uncertainty level defined according to the two-graph ROC and uncertainty interval methods on the testing data (*n* = 100)

Cutoff points represent two probability values of the ERG regression model that define the three levels of certainty. Sensitivity = a/(a + c). Specificity = d/(b + d). Accuracy: (a + d)/(a + b + c + d)

## Discussion

Prior work has revealed that ERG parameters provide a very accurate distinction between patients with schizophrenia and bipolar disorder compared to healthy controls [16]. This time our study proposes a simplified regression model that further supports the utility of the ERG as a biological instrument to monitor the risk of SMI (regrouping both schizophrenia and bipolar disorder). The results confirmed the very high accuracy and enhanced the efficiency of the clinical utility of the ERG by using only the cone ERG assessment which is less time consuming making the experience for the patient more comfortable.

The apparent performance of the predictive model showed very good discrimination, which remained robust after external validation using a testing dataset. Discriminative values after trichotomization (sensitivity = 0.91, specificity = 0.89, and accuracy = 0.90) and an AUC-ROC of 0.87 remained notably high, especially when compared to the other proposed biomarkers: event-related potential (accuracy = 0.79, sensitivity = 0.78, specificity =0.80) [40], electroencephalography (sensitivity = 0.89, specificity = 0.47) [41], blood-based laboratory test (sensitivity =0.83, specificity =0.83, AUC-ROC = 0.89) [42], eye movement abnormalities (accuracy = 0.98) [43]. The results also outperformed other detection instruments based on symptomatology, such as CARMS and SIPS, for which the pooled sensitivity and specificity estimates were 0.66 and 0.73, respectively, as reported by a meta-analysis [44]. Compared to all above instruments, the easiness and speed of recording of the ERG make it a strong candidate for clinical use.

The potential of photopic ERG as a biomarker has also been supported by other publications that reported significant differences in cone functions between SMI patients and healthy controls [13, 45, 46], although the protocols of ERG measurements may differ across studies. In addition, there is growing evidence suggesting that structural and functional retinal changes may reflect progressive brain neurodegeneration in mental disorders [10, 11, 47], as seen in multiple sclerosis and Alzheimer’s disease [48–50].

This study also provides insights into the clinical utility of the ERG as an instrument that can be used for decision-making (e.g., monitor the risk of SMI or offer intervention). ERG’s net benefit remained superior to the extreme hypothetical scenario of assuming all participants are positive for SMI and hence require further intervention. The superiority was evident for a range of threshold probabilities between 0.14–0.95. However, there are some limitations with this technique. First, there is no “gold standard” approach yet to compare the decision curves of Fig. 1, and the use of decision curve analysis is still very new to psychiatric research, which could explain the absence of other potential biomarkers assessed with this technique. Thus, the decision curve analysis presented in this study should be interpreted cautiously as an illustration of the potential value of our model. An intervention based on this type of decision tool still needs further investigation before reaching clear decision guidelines. Second, the decision curve analysis relies on the prevalence of the disease of interest [51], meaning that future research targeting a different population at an earlier stage of the disease is expected to have a different prevalence, which will have an important impact on the resulting decision curves.

Another limitation is the cross-sectional nature of this study which, despite providing valuable support for the development of ERG as a biomarker, is still considered as a preclinical exploratory phase in the sense that the present model predicts group membership (SMI patients or health subjects) rather than future development of SMI. Finally, overfitting was a central preoccupation; however, external validation was attempted using a testing dataset, and the results remained robust. Future longitudinal studies and replication in different samples are needed to address all the limitations cited above.

Nevertheless, the main strength of this study is the large sample of 501 participants (301 with SMI and 200 healthy controls); this allowed us to obtain more precise estimations and capture the diversity of the population. Another major strength is the introduction of uncertainty in the diagnostic levels. It is well known that a psychiatric diagnosis carries a social stigma that results in more issues to mental health and more functional impairment. The uncertainty level is represented here by an intermediate zone where the prediction values are not precise enough to make a diagnosis. Therefore, it allows practitioners to make a clinical decision about the next course of action with minimal misclassification rates and improves the accuracy or the correct classifications. In other words, it provides an option to offer further monitoring to inconclusive patients without the burden of a psychiatric stigma and increases diagnostic confidence.

## Conclusion

The ERG predicted SMI risk with a high level of accuracy when uncertainty was accounted for. Given that ERG is a noninvasive instrument already available in clinical settings and that a short photopic protocol may be sufficient, it could have the potential to become a useful clinical decision tool to intervene among at-risk subjects. Nevertheless, details on the moment of introduction during the developmental trajectory of SMI and the corresponding type of clinical decision need to be further investigated in longitudinal cohorts.

## Supplementary Information

**Additional file 1.**

## Data Availability

All data generated or analysed during this study are included in this article and its supplementary information files, further details are available from the corresponding author on reasonable request.

## Abbreviations

ERG: Electroretinogram
SMI: Severe mental illness
CAARMS: Comprehensive Assessment of at-Risk Mental States
SIPS: Structured Interview for Prodromal Syndromes
Amp: Amplitude
Lat: Latency
AUC-ROC: Area under the curve ROC
TG-ROC: Two graph ROC

## References

[1] Larsen TK, Melle I, Auestad B, Haahr U, Joa I, Johannessen JO (2011). Early detection of psychosis: positive effects on 5-year outcome. Psychol Med.

[2] Fusar-Poli P, McGorry PD, Kane JM (2017). Improving outcomes of first-episode psychosis: an overview. World Psychiatry.

[3] Van Der Gaag M, Smit F, Bechdolf A, French P, Linszen DH, Yung AR (2013). Preventing a first episode of psychosis: Meta-analysis of randomized controlled prevention trials of 12month and longer-term follow-ups. Schizophr Res [Internet].

[4] Yung AR, Yuen HP, McGorry PD, Phillips LJ, Kelly D, Dell’Olio M (2005). Mapping the onset of psychosis: the comprehensive assessment of at-risk mental states. Aust N Z J Psychiatry.

[5] Miller TJ, McGlashan TH, Rosen JL, Cadenhead K, Cannon T, Ventura J, et al. Prodromal assessment with the structured interview for prodromal syndromes and the scale of prodromal symptoms: predictive validity, interrater reliability, and training to reliability. Schizophr Bull 2004/03/03. 2003;29(4):703–715. 10.1093/oxfordjournals.schbul.a007040 14989408

[6] Fusar-poli P, Bonoldi I, Yung AR, Borgwardt S, Kempton MJ, Valmaggia L (2015). Predicting psychosis Meta-analysis of transition outcomes in individuals at high clinical risk. Arch Gen Psychiatry.

[7] Rutigliano G, Valmaggia L, Landi P, Frascarelli M, Cappucciati M, Sear V (2016). Persistence or recurrence of non-psychotic comorbid mental disorders associated with 6-year poor functional outcomes in patients at ultra high risk for psychosis. J Affect Disord [Internet].

[8] Chan MK, Cooper JD, Bahn S (2015). Commercialisation of biomarker tests for mental illnesses: advances and obstacles. Trends Biotechnol [Internet].

[9] Duffy A, Malhi GS, Grof P (2017). Do the trajectories of bipolar disorder and schizophrenia follow a universal staging model?. Can J Psychiatr.

[10] Almonte MT, Capellan P, Yap TE, Cordeiro MF (2020). Retinal correlates of psychiatric disorders. Ther Adv Chronic Dis.

[11] Silverstein SM, Rosen R (2015). Schizophrenia and the eye. Schizophr Res Cogn.

[12] Purves D, Augustine G, Fitzpatrick D, Katz L, LaMantia A, McNamara J, et al. The Retina. In: Sunderland (MA): Associates S, editor. Neuroscience [Internet]. 2 nd. 2001. Available from: https://www.ncbi.nlm.nih.gov/books/NBK10885/.

[13] Demmin DL, Davis Q, Roché M, Silverstein SM (2018). Electroretinographic anomalies in schizophrenia. J Abnorm Psychol.

[14] Gerbaldo H, Thaker G, Tittel PG, Layne-Gedge J, Moran M, Demisch L (1992). Abnormal electroretinography in schizophrenic patients with a history of sun gazing. Neuropsychobiology..

[15] Marmor MF, Hock P, Schechter G, Pfefferbaum A, Berger PA, Maurice R (1988). Oscillatory potentials as a marker for dopaminergic disease. Doc Ophthalmol.

[16] Hébert M, Mérette C, Gagné AM, Paccalet T, Moreau I, Lavoie J (2020). The Electroretinogram may differentiate schizophrenia from bipolar disorder. Biol Psychiatry [Internet].

[17] Gagné AM, Moreau I, St-Amour I, Marquet P, Maziade M. Retinal function anomalies in young offspring at genetic risk of schizophrenia and mood disorder: the meaning for the illness pathophysiology. Schizophr Res [Internet]. 2020:219(xxxx):19–24. Available from:. 10.1016/j.schres.2019.06.021. 10.1016/j.schres.2019.06.021 31320175

[18] Peredo R, Gagné AM, Gilbert E, Hébert M, Maziade M, Mérette C. Electroretinography may reveal cognitive impairment among a cohort of subjects at risk of a major psychiatric disorder. Psychiatry Res [Internet] 2020;291(April):113227. Available from: 10.1016/j.psychres.2020.113227. 10.1016/j.psychres.2020.113227 32593852

[19] Bellivier F, Geoffroy PA, Scott J, Schufhoff F, Leboyer M, Etain B. Biomarkers of bipolar disorder: specific or shared with schizophrenia? Frank. Front Biosci [Internet]. 2013:845–63 Available from: https://www.ncbi.nlm.nih.gov/pmc/articles/PMC5127822/. 10.2741/e665 PMC5127822 23747901

[20] Smeland OB, Bahrami S, Frei O, Savage J, Watanabe K, Krull F, et al. Genome-wide analysis reveals extensive genetic overlap between schizophrenia, bipolar disorder and intelligence. Mol Psychiatry. 2020. 10.1038/s41380-018-0332-x PMC6609490 30610197

[21] Vickers AJ, Cronin AM, Elkin EB, Gonen M (2008). Extensions to decision curve analysis, a novel method for evaluating diagnostic tests, prediction models and molecular markers. BMC Med Inform Decis Mak.

[22] Brǔlé J, Lavoie MP, Casanova C, Lachapelle P, Hébert M (2007). Evidence of a possible impact of the menstrual cycle on the reproducibility of scotopic ERGs in women. Doc Ophthalmol.

[23] Gagné AM, Lavoie J, Lavoie MP, Sasseville A, Charron MC, Hébert M (2010). Assessing the impact of non-dilating the eye on full-field electroretinogram and standard flash response. Doc Ophthalmol.

[24] Hébert M, Lachapelle P, Dumont M. Reproducibility of electroretinograms recorded with DTL electrodes. Doc Ophthalmol [Internet] 1995;91(4):333–342. Available from: 10.1007/BF01214651. 10.1007/BF01214651 8899303

[25] Hébert M, Vaegan LP (1999). Reproducibility of ERG responses obtained with the DTL electrode. Vis Res.

[26] Hébert M, Mérette C, Paccalet T, Gagné AM, Maziade M. Electroretinographic anomalies in medicated and drug free patients with major depression: tagging the developmental roots of major psychiatric disorders. Prog Neuro-Psychopharmacology Biol Psychiatry [Internet]. 2017;75:10–5. Available from:. 10.1016/j.pnpbp.2016.12.002. 10.1016/j.pnpbp.2016.12.002 28007463

[27] R Core Team (2020). R: a language and environment for statistical computing.

[28] Venables WN, Ripley BD (2002). Modern applied statistics with S [internet].

[29] Lavoie J, Gagné AM, Lavoie MP, Sasseville A, Charron MC, Hébert M (2010). Circadian variation in the electroretinogram and the presence of central melatonin. Doc Ophthalmol.

[30] Hébert M, Gagné AM, Paradis ME, Jomphe V, Roy MA, Mérette C (2010). Retinal response to light in young nonaffected offspring at high genetic risk of neuropsychiatric brain disorders. Biol Psychiatry [Internet]..

[31] Kuhn M. caret: Classification and Regression Training [Internet]. R package version 6.0–86; 2020. Available from: https://cran.r-project.org/package=caret.

[32] Zamanipoor Najafabadi AH, Ramspek CL, Dekker FW, Heus P, Hooft L, Moons KGM (2020). TRIPOD statement: a preliminary pre-post analysis of reporting and methods of prediction models. BMJ Open.

[33] TRIPOD. Assessing adherence of prediction model reports to the TRIPOD guideline 2015;(January):1–17.

[34] Steyerberg EW (2019). Clinical prediction models. Statistics for Biology and Health.

[35] Vickers AJ, Van Calster B, Steyerberg EW (2016). Net benefit approaches to the evaluation of prediction models, molecular markers, and diagnostic tests. BMJ..

[36] Van Calster B, Wynants L, Verbeek JFM, Verbakel JY, Christodoulou E, Vickers AJ (2018). Reporting and interpreting decision curve analysis: a guide for investigators. Eur Urol.

[37] Greiner M, Sohr D, Göbel P (1995). A modified ROC analysis for the selection of cut-off values and the definition of intermediate results of serodiagnostic tests. J Immunol Methods.

[38] Landsheer JA. Interval of uncertainty: an alternative approach for the determination of decision thresholds, with an illustrative application for the prediction of prostate cancer. PLoS One 2016;11(11):1–22. 10.1371/journal.pone.0166007 PMC5102386 27829010

[39] Vickers AJ, van Calster B, Steyerberg EW (2019). A simple, step-by-step guide to interpreting decision curve analysis. Diagnostic Progn Res.

[40] Johannesen JK, O’Donnell BF, Shekhar A, McGrew JH, Hetrick WP (2013). Diagnostic specificity of neurophysiological endophenotypes in schizophrenia and bipolar disorder. Schizophr Bull.

[41] Lenz D, Fischer S, Schadow J, Bogerts B, Herrmann CS (2011). Altered evoked gamma-band responses as a neurophysiological marker of schizophrenia?. Int J Psychophysiol [Internet].

[42] Schwarz E, Izmailov R, Spain M, Barnes A, Mapes JP, Guest PC (2010). Validation of a blood-based laboratory test to aid in the confirmation of a diagnosis of schizophrenia. Biomark Insights.

[43] Benson PJ, Beedie SA, Shephard E, Giegling I, Rujescu D, St. Clair D. (2012). Simple viewing tests can detect eye movement abnormalities that distinguish schizophrenia cases from controls with exceptional accuracy. Biol Psychiatry [Internet]..

[44] Chuma J, Mahadun P (2011). Predicting the development of schizophrenia in high-risk populations: systematic review of the predictive validity of prodromal criteria. Br J Psychiatry.

[45] Moghimi P, Torres-Jimenez N, McLoon L, Netoff T, Lee M, McDonald A (2020). Electoretinographic evidence of retinal ganglion cell-dependent function in schizophrenia. Schizophr Res.

[46] Balogh Z, Benedek G, Kéri S (2008). Retinal dysfunctions in schizophrenia. Prog Neuro-Psychopharmacology Biol Psychiatry.

[47] Lizano P, Bannai D, Lutz O, Kim LA, Miller J, Keshavan M (2020). A Meta-analysis of retinal Cytoarchitectural abnormalities in schizophrenia and bipolar disorder. Schizophr Bull.

[48] Ferrari L, Huang SC, Magnani G, Ambrosi A, Comi G, Leocani L (2017). Optical coherence tomography reveals retinal Neuroaxonal thinning in frontotemporal dementia as in Alzheimer’s disease. J Alzheimers Dis.

[49] Siger M, Dziȩgielewski K, Jasek L, Bieniek M, Nicpan A, Nawrocki J (2008). Optical coherence tomography in multiple sclerosis: thickness of the retinal nerve fiber layer as a potential measure of axonal loss and brain atrophy. J Neurol.

[50] Liu D, Zhang L, Li Z, Zhang X, Wu Y, Yang H (2015). Thinner changes of the retinal nerve fiber layer in patients with mild cognitive impairment and Alzheimer’s disease. BMC Neurol.

[51] Pulleyblank R, Chuma J, Gilbody SM, Thompson C (2013). Decision curve analysis for assessing the usefulness of tests for making decisions to treat: an application to tests for prodromal psychosis. Psychol Assess.

