# Supplementary material for: Developing a clinical decision tool based on electroretinogram to monitor the risk of severe mental illness
Source: BMC Psychiatry. 2022 Nov 18;22:718. doi: 10.1186/s12888-022-04375-3 (PMC9673390; doi:10.1186/s12888-022-04375-3)
Supplement: Supplementary file 1 — Additional file 1. [file 12888_2022_4375_MOESM1_ESM.docx]

**Supplements**

Figure S1: Area under a ROC Curve (AUC-ROC). Cones ERG only model and Cones+Rods ERG model.


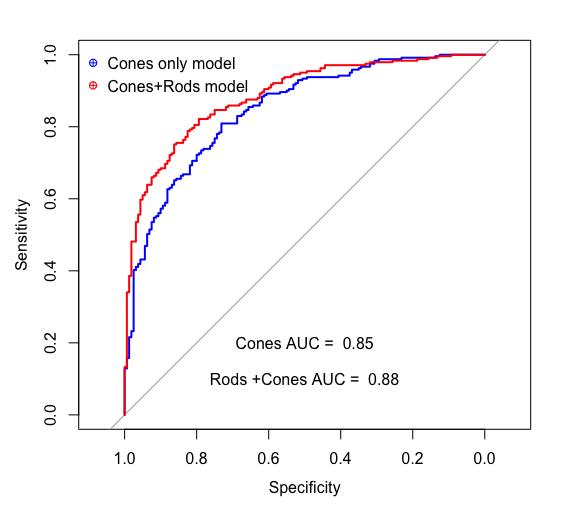


**Table S1: Unadjusted association between each candidate predictor (ERG measurements) and the outcome (SMI).**

| **Predictors** | **Intercept** | **Coefficient**  **Estimate** | **SE** | **p-value** | **Goodness of fit** | |
| --- | --- | --- | --- | --- | --- | --- |
|  |  |  |  |  | **BIC** | **AIC** |
| a-wave amplitude |  |  |  |  |  |  |
| Fixed | 1.79 | -0.09 | 0.02 | <0.001* | 665.02 | 656.59 |
| Vmax | 2.96 | -0.11 | 0.02 | <0.001* | 649.82 | 641.38 |
|  |  |  |  |  |  |  |
| a-wave latency |  |  |  |  |  |  |
| Fixed | 1.35 | -0.06 | 0.06 | 0.340 | 685.55 | 677.12 |
| Vmax | 2.14 | -0.12 | 0.10 | 0.216 | 684.92 | 676.49 |
|  |  |  |  |  |  |  |
| b-wave amplitude |  |  |  |  |  |  |
| Fixed | 1.03 | -0.01 | 0.00 | 0.058 | 682.86 | 674.42 |
| Vmax | -32.12 | 1.02 | 0.10 | <0.001* | 532.79 | 524.36 |
|  |  |  |  |  |  |  |
| b-wave latency |  |  |  |  |  |  |
| Fixed | -22.31 | 0.79 | 0.09 | <0.001* | 584.94 | 576.51 |
| Vmax | 1.82 | -0.02 | 0.01 | <0.001* | 674.74 | 666.31 |

**Table S2. Full logistic regression model (all ERG variables) on the training dataset (n=401).**

| **Predictors** | **Model coefficient**  **Estimate** | **SE** | **p-value** | **Goodness of fit** | | **AUC** |
| --- | --- | --- | --- | --- | --- | --- |
|  |  |  |  | BIC | AIC |  |
| Intercept | 31.28 | 4.08 | <0.001 | 450.95 | 399.03 | 0.85 |
|  |  |  |  |  |  |  |
| a-wave amplitude |  |  |  |  |  |  |
| Fixed | -0.07 | 0.04 | 0.04 |  |  |  |
| Vmax | -0.09 | 0.04 | 0.02 |  |  |  |
|  |  |  |  |  |  |  |
| a-wave latency |  |  |  |  |  |  |
| Fixed | 0.05 | 0.10 | 0.631 |  |  |  |
| Vmax | -0.62 | 0.17 | <0.001 |  |  |  |
|  |  |  |  |  |  |  |
| b-wave amplitude |  |  |  |  |  |  |
| Fixed | 0.01 | 0.02 | 0.64 |  |  |  |
| Vmax | 0.01 | 0.02 | 0.48 |  |  |  |
|  |  |  |  |  |  |  |
| b-wave latency |  |  |  |  |  |  |
| Fixed | 0.27 | 0.18 | 0.13 |  |  |  |
| Vmax | 1.08 | 0.18 | <0.001 |  |  |  |
|  |  |  |  |  |  |  |
| Age | -0.02 | 0.01 | 0.13 |  |  |  |
|  |  |  |  |  |  |  |
| Sex (Male) | -0.56 | 0.25 | 0.03 |  |  |  |
|  |  |  |  |  |  |  |
| Pupil size | 0.14 | 0.11 | 0.22 |  |  |  |

**Table S3. Sensitivity model (medication added as a potential covariate to the final best model). On training dataset (n = 401).**

| **Predictors** | **Model coefficient**  **Estimate** | **SE** | **p-value** |  | **Goodness of fit** | | **Discriminative ability** | |
| --- | --- | --- | --- | --- | --- | --- | --- | --- |
|  |  |  |  |  | **BIC** | **AIC** | **Accuracy** | **AUC** |
| Intercept | 5.97 | 1.58 | 0.997 |  | 256.18 | 229 | 0.77 | 0.85 |
|  |  |  |  |  |  |  |  |  |
| a-wave amplitude Fixed | 0.01 | 0.04 | 0.820 |  |  |  |  |  |
|  |  |  |  |  |  |  |  |  |
| b-wave latency  Fixed | -0.34 | 0.26 | 0.178 |  |  |  |  |  |
|  |  |  |  |  |  |  |  |  |
| b-wave latency  Vmax | 1.40 | 0.28 | <0.001 |  |  |  |  |  |
|  |  |  |  |  |  |  |  |  |
| Age | -0.06 | 0.02 | 0.001 |  |  |  |  |  |
|  |  |  |  |  |  |  |  |  |
| Sex (Male) | -0.12 | 0.37 | 0.736 |  |  |  |  |  |
|  |  |  |  |  |  |  |  |  |
| Medication | -1.93 | 7.89 | 0.980 |  |  |  |  |  |
